# Malaria prevention during pregnancy: Implications for maternal and neonatal health in East Africa

**DOI:** 10.1016/j.nmni.2023.101186

**Published:** 2023-10-06

**Authors:** Kanza Farhan, Navira Saeed, Sufyan Raza Khan, Burhan Tariq, Aliza Ahmed, Aymar Akilimali

**Affiliations:** Jinnah Sindh Medical University, Karachi, Pakistan; Department of Research, Medical Research Circle (MedReC), Bukavu, Democratic Republic of the Congo

*Dear Editor,*

Globally more than 125 million women are at risk of contracting malaria during pregnancy due to their weakened cell mediated immunity and glucose metabolism [1]. In sub-Saharan Africa's endemic areas, Malaria in pregnancy (MIP) is a significant prevalent cause of maternal mortality and adverse newborn outcomes. The World Health Organization (WHO)'s World Malaria Report estimates that 241 million cases and 627,000 fatalities were caused by malaria in 2020. Africa accounts for 93 ​% of all malaria deaths worldwide [2]. As a researcher the high prevalence of malaria in the continent, which disproportionately affects pregnant women, makes it imperative to address malaria-related maternal deaths. This also increases the burden on the region's inadequate infrastructure, limited access to medical services in rural areas, and inadequate healthcare funding which is already suffering after Covid-19 and recent Cholera, Ebola and Marburg virus outbreaks in the region. *Plasmodium falciparum* and *Plasmodium vivax*, are known to cause serious problems for pregnant women and their unborn children. P. falciparum is the predominant species in sub-Saharan Africa, where a significant number of unfavorable birth outcomes are linked to malarial occurrence [1]. The entire East African Community (EAC), which includes the Republics of Kenya, Uganda, Rwanda, Burundi, and Tanzania, has been affected by rising temperatures as well as an increase in the frequency and severity of extreme weather events as a result of regional and global climate change. This has led to the spread of malaria into regions that had not previously been exposed to the disease, together with rising parasite resistance to medication and a reduction in funding for vector management [3].

Congenital malaria, maternal anemia, stillbirth, preterm birth, maternal and neonatal mortality, and low birth weight (defined as birth weight <2.5 kg) are some of the adverse effects of malaria that harm both the mother and the newborn [4]. According to the WHO, there were an anticipated 33 million births in 33 countries with moderate to high malaria transmission in 2019. An estimated 822,000 newborns were born as a result of malaria infection during the pregnancy, low birth weight (LBW) was present in 35 ​% of these births. In agreement with this, it has been estimated that LBW related to malaria infections during pregnancy causes 11 ​% of neonatal mortality in sub-Saharan Africa [5].

Malaria remains a significant public health issue in East Africa due to challenges such as access to healthcare, infrastructure limitations, social beliefs, and lack of awareness. Hospital mortality due to severe malaria is high, and there are disparities in access to antenatal clinics (ANC) between urban and rural populations. *P. Falciparum* malaria in pregnancy can easily be reduced by integrating malarial prevention techniques with maternal and neonatal healthcare services, as over 70 ​% of women in Africa attend antenatal clinics at least once during pregnancy [4]. The WHO recommends a three-pronged strategy for control of malaria in pregnancy in Africa, including case management, use of insecticide-treated nets (ITNs), and intermittent preventive treatment (IPTp) in areas with stable transmission and high ANC attendance. Community-based programs are needed in areas with low antenatal coverage [3].

The pillars of African society must work together to combat malaria. In situations when there are technical or budgetary constraints, collaborative efforts of government and NGOs are required for malaria prevention. Understanding a community's perception of febrile disease, its role in people's belief systems about sickness, and current behaviors that can help or impede preventive actions, is substantial [2]. The success of Switzerland in creating anti-malarial medications is evidence of productive cooperation between citizens, NGOs, and the government. Hence *Plasmodium falciparum* malaria during pregnancy is a prominent contributor to maternal and newborn mortality rates in African regions. Despite significant progress in recent years, there remain several problems, shortcomings, and room for improvement in this field. These issues are of utmost importance and urgency in order to achieve the global standards set by the WHO for the eradication and prevention of malaria and to ensure improved healthcare quality for pregnant women specifically. Immediate, concerned and collaborative efforts are necessary to enhance preventive measures, raise awareness, eliminate misconceptions, and foster collective action in safeguarding the well-being of women and children, probably the most vulnerable sections of African society [1].

## Ethics approval and consent to participle

Not applicable.

## Consent for publication

Not applicable.

## Availability of data and material

Not applicable.

## Funding

The authors did not receive any financial support for this work. No funding has been received for the conduct of this study.

## Authors’ contributions

Conception***:*** Kanza FARHAN and Burhan TARIQ***, Design:*** Aymar AKILIMALI***, Project administrator;*** Burhan TARIQ and Aliza AHMED***, Supervisor:*** Kanza FARHAN, Funding ***acquisition;*** Kanza FARHAN, ***Investigation;*** Aymar AKILIMALI, ***Resources;*** Kanza FARHAN, Burhan TARIQ and Aliza AHMED, ***Validation;*** Aymar AKILIMALI, ***Visualization;*** Kanza FARHAN***, Literature search:*** Aymar AKILIMALI and Kanza FARHAN***, Manuscript preparation:*** Navira SAEED, Sufyan Raza KHAN***, Manuscript editing;*** Aliza AHMED, Burhan TARIQ and Aymar AKILIMALI***, Manuscript review:*** All authors***, Final approval of manuscript***: All Authors.

## Provenance and peer review

Not commissioned, externally peer reviewed.

## Declaration of competing interest

The authors declare that there no conflict of interest.
